# “K Cramps,” Recurrent Abdominal Pain in a Patient with Chronic Ketamine Use: A Case Report

**DOI:** 10.5811/cpcem.19431

**Published:** 2024-08-01

**Authors:** Tucker Avra, Jesus Torres, Kumar Felipe Vasudevan, Elizabeth A. Samuels

**Affiliations:** * UCLA David Geffen School of Medicine, Los Angeles, California; † UCLA David Geffen School of Medicine, Department of Emergency Medicine, Los Angeles, California; ‡ UCLA David Geffen School of Medicine, Department of Internal Medicine, Los Angeles, California

**Keywords:** *ketamine*, *case report*, *harm reduction*, *substance use disorder*, *ketamine use disorder*

## Abstract

**Introduction:**

Medical and nonmedical ketamine use is increasing in the United States. This will likely lead to an increase in emergency department (ED) visits in individuals experiencing associated side effects. Physicians will need to be able to effectively recognize and manage ketamine-related complications.

**Case Report:**

A 31-year-old male with a three-year history of inhalational, intramuscular, and intravenous nonmedical ketamine use presented to the ED twice within a week with symptoms of severe atraumatic back pain, abdominal pain, and dyspepsia. A comprehensive workup, including advanced imaging, was unrevealing for identifiable causes, and the patient was discharged with instructions for primary care follow-up for further evaluation. The patient used information shared on Reddit, an online forum and social network, to identify that the cause of his pain was related to chronic ketamine use. Subsequently, upon discontinuation of ketamine, the pain improved in 24 hours. The patient self-navigated to addiction treatment.

**Conclusion:**

Emergency physicians should consider sequelae of chronic ketamine use as a possible cause for gastrointestinal and urologic symptoms in the ED. In addition to thorough examination and assessment for other acute medical problems, patients should be offered education, symptomatic treatment, and linkage to harm reduction and substance use disorder treatment services.

CPC-EM Capsule What do we already know about this clinical entity?

*Abdominal pain is a common complication from chronic ketamine use, but underlying mechanisms are poorly understood.*

What makes this presentation of disease reportable?

*This is the first reported case of abdominal pain associated with ketamine use in the United States peer-reviewed literature.*

What is the major learning point?

*Gastrointestinal side effects should be on the differential when assessing abdominal pain in individuals who use ketamine.*

How might this improve emergency medicine practice?

*With increases in ketamine use, emergency physicians will need to be able to recognize and care for patients with non-neurologic complications of chronic ketamine use.*

## INTRODUCTION

Ketamine is becoming increasingly used in the United States (US) for a variety of mental health conditions, most notably treatment-resistant depression.^1^ Intranasal esketamine (S-enantiomer of ketamine) was approved in 2019 by the US Food and Drug Administration (FDA) for treatment-resistant depression. There is also widespread off-label medical use of intravenous (IV) ketamine for other mental health conditions, including post-traumatic stress disorder, anxiety, eating disorders, and mood disorders.

Ketamine is also used recreationally for its dissociative and psychedelic effects. Over the last two decades, self-reported, nonmedical ketamine use and exposures in the US have been gradually increasing, and seizures of illicit ketamine increased 349% from 2017 to 2022.^2^ As rates of recreational and medical use of ketamine rise, physicians need to know how to recognize and treat side effects and complications of ketamine use. However, there is a paucity of US literature discussing how to identify and manage side effects of chronic ketamine use. Studies in the United Kingdom, Hong Kong, and Italy describe a variety of clinical presentations in people with chronic ketamine use, including urological, neurological, and gastrointestinal (GI) symptoms.^3^ ^,^ ^4^ Common presentations of these symptoms include dysuria for urological symptoms, intoxication or hallucinations for neurological symptoms, and abdominal pain for GI symptoms. In this case report, we describe clinical presentation and management of recurrent severe abdominal pain—known informally as “K cramps” in the community of people who use ketamine—associated with long-term use.

## CASE REPORT

A 31-year-old male with a history of chronic ketamine use, major depressive disorder, and chronic back pain presented to the emergency department (ED) with two days of increasingly severe back pain and chest tightness. He had a similar episode of back pain and chest tightness eight weeks prior that lasted for 10 days, with no other symptoms. He did not seek medical treatment for that episode, and the pain slowly resolved on its own. The patient took intramuscular and IV ketamine daily for eight months, with each day’s doses ranging from 1–3 grams. Before intramuscular and IV use, he had been insufflating 0.5–1 gram every day for three years.

At presentation to the ED, he was afebrile, heart rate of 77 beats per minute, blood pressure of 131/86 millimeters of mercury, and an oxygen saturation of 100% on room air. He was awake and alert and in mild distress. Physical examination revealed mild tenderness on thoracic paraspinal muscle palpation, mild tenderness to palpation in the left upper quadrant, and was otherwise unremarkable. Complete blood count, complete metabolic panel, urinalysis, electrocardiogram, troponin, and D-dimer were within normal limits. Chest radiographs were negative for pneumonia, pneumothorax, pneumomediastinum, or subcutaneous air. Given back pain and injection use, a thoracic spine magnetic resonance imaging (MRI) was performed to rule out an epidural abscess. The MRI revealed no infection, fluid collection, disc disease, fractures, or other acute abnormality that could account for his symptoms. Two sets of blood culture were negative. The patient was treated with acetaminophen and discharged with instructions to continue acetaminophen and ibuprofen at home for pain management.

The following day, the pain migrated to his upper abdomen and he developed dyspepsia. He reported taking famotidine, calcium carbonate, acetaminophen, and ibuprofen daily, with no improvement in symptoms. He continued daily ketamine use for recreational purposes and to control the pain. Every night, the pain woke him up at 4 am. The pain, which the patient described as a deep aching/cramping sensation, was worse after eating and improved with fasting. The intensity remained severe, and he returned to the ED five days later for reevaluation.

On the second ED visit, the patient reported 9/10 abdominal pain on arrival. Again, vital signs showed no abnormalities. He was awake and alert and in moderate distress. Physical examination revealed right lower quadrant tenderness to palpation and mild right upper quadrant tenderness.

He was treated with 4 milligrams IV morphine and 1 liter lactated Ringer’s IV fluid. The pain improved to an 8/10 and the morphine was repeated two hours later, resulting in 6/10 abdominal pain. Computed tomography of the abdomen and pelvis with contrast was performed and revealed no evidence of an acute intra-abdominal process. A complete abdominal ultrasound was performed, revealing a 2–3 mm adherent gallstone and a 3 mm gallbladder polyp. The patient was discharged with instructions to continue ibuprofen and acetaminophen, follow up with primary care, and return to the ED in two days if there was no improvement.

After discharge, the patient searched for his symptoms on Reddit, where he discovered several discussion forums about “K Cramps/K Pains,” Reddit users describe the pain as feeling like someone was squeezing their organs as hard as they could, pain that reaches down into the upper abdomen and creeps though the chest, and feeling like a hot knife was running through the back to the front of the stomach. He followed peer recommendations to take hot baths and discontinue ketamine. After discontinuing ketamine, the pain improved within 24 hours. He restarted recreational ketamine 72 hours later, with initial recurrence of dyspepsia four hours after use. The following day he developed the severe abdominal pain again, which lasted for two days until he discontinued ketamine again and admitted himself to a residential substance use disorder (SUD) treatment program. The pain improved 30 hours after starting SUD treatment. The patient communicated this information to his ED treatment team during his residential treatment stay.

## DISCUSSION

We report a case of recurrent abdominal pain associated with chronic high doses of ketamine, colloquially known as “K cramps.” The patient initially presented with reported back pain and chest tightness with abdominal tenderness on physical exam that progressed to severe upper abdominal pain. Diagnostics ruled out infection or other life-threatening causes of his symptoms, and the patient was discharged with instructions to continue acetaminophen and ibuprofen and return in two days if the pain had not improved. The patient was open about his ketamine use to the emergency physicians. However, outside of possible epidural abscess or infection due to at-home IV and intramuscular ketamine use, there was no consideration of the possible association of symptoms as a side effect or complication of ketamine use. In addition, there was no recommendation or counseling to reduce use, harm reduction counseling, motivational interviewing, or screening, brief intervention, and referral to treatment.

Cross-sectional studies have described the most common toxicity patterns associated with ketamine, primarily presenting with neurological, urological, and gastrointestinal symptoms.^3^ A review of 233 ED visits among people who use ketamine in Hong Kong showed that the most common presenting symptoms were altered consciousness (45%), abdominal pain (21%), urinary symptoms (12%), and dizziness (12%) (Table).^6^ In addition, many forums on the internet describe and verify abdominal pain as a common symptom manifestation for patients.

**Table.** Clinical presentations, mechanisms of action, and treatment of side effects associated with ketamine use.

System affected	Symptom(s)	Findings	Mechanism of action	Treatment
Neurologic	Intoxication Altered mental status Agitation Muscle rigidity Hallucinations Dissociative emergent reactions	Clinical observation	NMDA-receptor antagonism	Benzodiazepines
Gastrointestinal	Abdominal pain	Cholestasis Acalculous biliary colic Common bile duct dilation Abnormal liver function tests Gastritis Gastroduodenitis	Unknown	Hot baths Hot beverages Cessation of ketamine use
Urologic	Dysuria Urinary urgency Hematuria	Sterile urine Ulcerative cystitis Obstructive nephropathy	Direct damage to urothelial lining by ketamine and ketamine metabolites	Cessation of ketamine use

*NMDA*, N-methyl-D-aspartate.

“K Cramps” is mentioned only one time in peer-reviewed literature, described as “severe abdominal pains” that “can feel like severe gas pains but much worse.”^5^ In one cross-sectional study of patients seeking treatment for ketamine uropathy, 27.5% reported upper GI symptoms, including epigastric pain and recurrent vomiting.^4^ Some case reports indicate the pain is biliary or gastric in etiology. Biliary causes include cholestasis, chronic acalculous biliary colic, and common bile duct dilation.^7^ ^,^ ^8^ Abnormal liver function tests have also been associated with therapeutic ketamine use, and the mechanism is unknown.^9^ ^,^ ^10^ Poon et al evaluated GI problems among individuals with inhalational ketamine use. Among 28 patients with GI symptoms, 14 patients had endoscopies, with *Helicobacter pylori*-negative gastritis as the most common histopathological finding, followed by gastroduodenitis.^9^ The only reported treatment for ketamine-associated abdominal pain in the peer-reviewed literature is abstinence, although Reddit threads reveal numerous supportive measures, including hot showers and baths, hot beverages, and small meals, to support patients in managing their pain.

In addition to abdominal pain, emergency physicians should be familiar with the severe neurologic and urologic complications of chronic ketamine use. Patients may experience altered mental status, agitation, muscle rigidity, and dissociative emergence reactions that may include hallucinations, fear, and “out of body” experiences.^3^ Benzodiazepines are the primary treatment for dissociative emergence reactions, muscle rigidity, and agitation due to ketamine use.^11^ The urological side effects of ketamine use are well described and reportedly affect approximately one-third of chronic ketamine users.^12^ Clinical symptoms of urological toxicity are dysuria, urinary urgency, and possible progression to painful hematuria. These symptoms are associated with ulcerative cystitis secondary to ketamine and its metabolites that may progress to obstructive nephropathy and bladder fibrosis.^13^ ^,^ ^14^ Abstinence of ketamine use is described as the only treatment, with no reports of resolution of urological symptoms with persistent ketamine use (Table).^12^

There are limited case reports in the US of clinical presentations associated with ketamine toxicity. Ketamine side effects should be on the differential when assessing abdominal pain and dysuria in individuals who admit to ketamine use. However, it is a diagnosis of exclusion after other emergent or infectious causes are appropriately ruled out. While no pharmacotherapy has been approved by the FDA for ketamine use disorder, primary counseling to address symptoms should focus on reduction or cessation of ketamine use, including motivational interviewing and referral and linkage to addiction treatment. For all patients who inject drugs, emergency physicians should be familiar with and provide resources on sterile consumption equipment and safer injection technique for infection prevention. Furthermore, given the frequent presence of fentanyl adulterants in the drug supply, all patients who use drugs should be offered take-home naloxone (or naloxone prescription) and fentanyl test strips for overdose prevention.^15^

## CONCLUSION

In this case, lack of clinician knowledge about “K cramps” contributed to delayed addiction treatment engagement and resolution of symptoms. The patient relied on advice and input from an online forum of people who use or have used ketamine to self-guide his treatment. Given that the patient reported using 1–3 grams of ketamine daily he should have been referred for addiction treatment. People who use drugs have historically, and currently, developed—and subsequently tested—strategies to reduce morbidity and mortality among themselves and their peers. This has included harm reduction strategies such as syringe services programs, naloxone distribution, drug checking initiatives, and peer support and information sharing. As nonmedical ketamine use increases in the US, emergency physicians may see more patients with symptoms and complications related to chronic use. Further work is needed to understand the etiology and treatment of GI symptoms related to ketamine use, which should include being aware of the knowledge and perspectives of people who have used ketamine and testing the strategies they have developed to treat GI symptoms.
